# Identifying potential biomarkers of idiopathic pulmonary fibrosis through machine learning analysis

**DOI:** 10.1038/s41598-023-43834-z

**Published:** 2023-10-02

**Authors:** Zenan Wu, Huan Chen, Shiwen Ke, Lisha Mo, Mingliang Qiu, Guoshuang Zhu, Wei Zhu, Liangji Liu

**Affiliations:** 1 The Clinical Medical School, Jiangxi University of Traditional Chinese Medicine, Nanchang, Jiangxi China; 2 The Eighth Affiliated Hospital of Sun Yat-sen University, Shenzhen, China; 3 The Affiliated Hospital of Jiangxi University of Traditional Chinese Medicine, Nanchang, China; 4 The Second Clinical Medical School, Guangzhou University of Chinese Medicine, Guangzhou, Guangdong China

**Keywords:** Preclinical research, Biological techniques, Bioinformatics, Biological models

## Abstract

Idiopathic pulmonary fibrosis (IPF) is the most common and serious type of idiopathic interstitial pneumonia, characterized by chronic, progressive, and low survival rates, while unknown disease etiology. Until recently, patients with idiopathic pulmonary fibrosis have a poor prognosis, high mortality, and limited treatment options, due to the lack of effective early diagnostic and prognostic tools. Therefore, we aimed to identify biomarkers for idiopathic pulmonary fibrosis based on multiple machine-learning approaches and to evaluate the role of immune infiltration in the disease. The gene expression profile and its corresponding clinical data of idiopathic pulmonary fibrosis patients were downloaded from Gene Expression Omnibus (GEO) database. Next, the differentially expressed genes (DEGs) with the threshold of FDR < 0.05 and |log2 foldchange (FC)| > 0.585 were analyzed via R package “DESeq2” and GO enrichment and KEGG pathways were run in R software. Then, least absolute shrinkage and selection operator (LASSO) logistic regression, support vector machine-recursive feature elimination (SVM-RFE) and random forest (RF) algorithms were combined to screen the key potential biomarkers of idiopathic pulmonary fibrosis. The diagnostic performance of these biomarkers was evaluated through receiver operating characteristic (ROC) curves. Moreover, the CIBERSORT algorithm was employed to assess the infiltration of immune cells and the relationship between the infiltrating immune cells and the biomarkers. Finally, we sought to understand the potential pathogenic role of the biomarker (SLAIN1) in idiopathic pulmonary fibrosis using a mouse model and cellular model. A total of 3658 differentially expressed genes of idiopathic pulmonary fibrosis were identified, including 2359 upregulated genes and 1299 downregulated genes. FHL2, HPCAL1, RNF182, and SLAIN1 were identified as biomarkers of idiopathic pulmonary fibrosis using LASSO logistic regression, RF, and SVM-RFE algorithms. The ROC curves confirmed the predictive accuracy of these biomarkers both in the training set and test set. Immune cell infiltration analysis suggested that patients with idiopathic pulmonary fibrosis had a higher level of B cells memory, Plasma cells, T cells CD8, T cells follicular helper, T cells regulatory (Tregs), Macrophages M0, and Mast cells resting compared with the control group. Correlation analysis demonstrated that FHL2 was significantly associated with the infiltrating immune cells. qPCR and western blotting analysis suggested that SLAIN1 might be a signature for the diagnosis of idiopathic pulmonary fibrosis. In this study, we identified four potential biomarkers (FHL2, HPCAL1, RNF182, and SLAIN1) and evaluated the potential pathogenic role of SLAIN1 in idiopathic pulmonary fibrosis. These findings may have great significance in guiding the understanding of disease mechanisms and potential therapeutic targets in idiopathic pulmonary fibrosis.

## Introduction

Idiopathic pulmonary fibrosis (IPF) is a progressive, chronic, fibrotic interstitial lung disease with unknown etiology and poor prognosis^1, 2^. Idiopathic pulmonary fibrosis is characterized by lung tissue remodeling and scarring, decreased lung function, and decreased quality of life, which eventually leads to respiratory failure^3–7^. In the elderly, this disease is more prevalent and the average survival rate is only 2–3 years following diagnosis^8^. Despite the introduction of new antifibrotic therapies, idiopathic pulmonary fibrosis remains a fatal disease with a median survival of 5.7 years and limited treatment options^9, 10^. Therefore, it is urgent to find biomarkers for the treatment of idiopathic pulmonary fibrosis.

Machine learning is a data analysis method that automatically constructs analytical models, which has been widely used in clinical medicine^11^. Previous studies showed that machine learning can be used to predict myocardial infarction, identify pathologies, and improve surgical outcomes^12^. In recent years, machine learning has been widely used in the diagnosis and treatment of many diseases^13–15^. In addition, machine learning algorithms known as random forest (RF) models are made up of many individual decision trees that are constructed iteratively from random subsets of predictor and dependent variables^16^. The least absolute shrinkage and selection operator (LASSO) logistic regression analysis is a linear regression method with regularization that can be applied to high-dimensional analysis^17^. Support vector machine-recursive feature elimination (SVM-RFE) can be used to screen the best combinations of variables after modeling different numbers of variables due to its nonlinear discrimination characteristics^18^. Thus, using machine learning algorithms to identify biomarkers of idiopathic pulmonary fibrosis is of great significance.

In this study, we downloaded the gene expression profile and its corresponding clinical data of idiopathic pulmonary fibrosis patients from the GEO database. Based on this data, the DEGs with the threshold of FDR < 0.05 and |log_2_ foldchange (FC)|> 0.585 were identified between idiopathic pulmonary fibrosis and normal samples. Then, we combined least absolute shrinkage and selection operator (LASSO) logistic regression, support vector machine-recursive feature elimination (SVM-RFE), and random forest (RF) algorithms to screen out the biomarkers of idiopathic pulmonary fibrosis. The accuracy of these biomarkers was evaluated according to ROC curves. Moreover, we used the CIBERSORT algorithm to assess the infiltration of immune cells and the relationship between the infiltrating immune cells and the biomarkers. Finally, we assessed the potential pathogenic role of the biomarker (SLAIN1) in the development of idiopathic pulmonary fibrosis using mouse and cellular models.

## Methods

### Animals

C57BL6 mice aged 8 weeks were obtained from Jackson Laboratories, which were kept under specific pathogen-free (SPF) conditions in the animal barrier facility of Jiangxi University of Traditional Chinese Medicine. The study and all procedures were conducted in accordance with the ethical guidelines and regulations approved by the Ethics Committee of Jiangxi University of Traditional Chinese Medicine. We confirm that all methods were performed in accordance with the relevant guidelines and regulations (https://www.nature.com/srep/journal-policies/editorial-policies#experimental-subjects). Upon arrival, mice were acclimatized for 3 days before induction of idiopathic pulmonary fibrosis, and their health and wellbeing were monitored at least daily throughout the experiments. All experiments were conducted and reported according to ARRIVE guidelines (https://arriveguidelines.org/arrive-guidelines).

### Cell culture

Human fetal lung fibroblast cell line (HFL1) and the human pulmonary epithelial A549 cell line were purchased from the American Type Culture Collection (ATCC). A549 lung cancer cells were cultured in DMEM medium supplemented with 2mMl glutamine and 10% fetal bovine serum (FBS). HFL1 cells were cultured with RPMI 1640 medium supplemented with 2mMl glutamine and 10% fetal bovine serum (FBS). Both cell lines were incubated in a humidified incubator with 5% CO_2_ at 37 °C and supplemented with 1% penicillin and 1% streptomycin. TGF-β was used to produce a cellular model of pulmonary fibrosis in A549 and HFL1 for 48 h.

### Western blotting analysis

Collected lung tissue samples were cut into pieces, incubated with RIPA lysis buffer on ice for 40 min, and cleared by centrifugation at 13,000 rpm/min for 5 min. Protein concentrations were measured using the BCA Protein Assay kit (Beotime Institute of Biotechnology, Nanjing, China) according to the manufacturer's instructions. Then, 50 μg of protein from each sample was separated by 10% sodium dodecyl sulfate–polyacrylamide gel electrophoresis (SDS-PAGE) and transferred to a polyvinylidene fluoride (PVDF) membrane. Membranes were blocked with 5% skim milk or 5% bovine serum albumin (BSA) and incubated overnight at 4 °C with the indicated concentrations of the following primary antibodies from Abcam (Cambridge, UK): Actin (Cat. No. ab179467; dilution, 1:5000; Abcam) and SLAIN-1 Polyclonal antibody (Cat no: 22123-1-AP; dilution, 1:1000; Protein Tech Group, Inc). This was followed by incubation with secondary antibodies (dilution, 1:5000–1:10,000; Abcam) at 37 °C for 1 h. The labeled protein bands were scanned on an HP Scanjet 5500 (Hewlett Packard France, Les Ullis, France). Finally, the relative protein concentration was determined by the gray level of the band with the Quantity One 4.40 software (Bio-Rad Laboratories Inc).

### HE staining and Masson staining

Lung tissue was fixed with 4% polyformaldehyde solution for 24 h, dehydrated and paraffin embedded and cut into 4-μm slices. Then, the slices (3 slices per mouse) were stained with hematoxylin and eosin (H&E) to observe histopathological changes in the colon under a microscope. Masson trichrome staining was performed using Masson trichrome staining kit (Beyotime, China). Histological sections were stained with hematoxylin and eosin (H&E) to assess histopathological changes, and Masson trichrome staining was performed following the manufacturer's instructions.

### RNA isolation and quantitative real-time PCR (qPCR)

RNA was extracted from lung homogenates or cultured cells using Qiagen RNeasy kits or Trizol (Vazyme) according to the manufacturer's instructions. Reverse transcription was performed using the HiScript○R II Q RT SuperMix Kit (Vazyme, Nanjing, China) as instructed by the manufacturer. SYBR green (Vazyme) and Quantstudio 6 Real-Time PCR System (Applied Biosystems) were used for qPCR. GeneCopoeia provided the primers that were used in this study. The normalizer employed in this study was GAPDH. The following primers were used for qPCR: Human GAPDH: 5′-GGA GCG AGA TCC CTC CAA AAT-3′ and 5′-GGC TGT TGT CAT ACT TCT CAT GG-3′;Human SLAIN1: 5′-CAT CAC CGG GAC AGC TTC AA-3′ and 5′-GAA CGG TTG GAC TCA CAT AGG-3′;Mouse GAPDH: 5′- AGG TCG GTG TGA ACG GAT TTG-3′ and 5′- TGT AGA CCA TGT AGT TGA GGT CA -3′;Mouse Slain1: 5′- ACT GAT GTT CAG ATC ATG GCT CG-3′ and 5′- ACT GCA TGT CCC CTT TTT CCC-3′.

### Data acquisition

Data for idiopathic pulmonary fibrosis and healthy specimens were downloaded from the GEO dataset (GEO, https://www.ncbi.nlm.nih.gov/geo/database). The GSE150910 dataset, consisting of 103 idiopathic pulmonary fibrosis samples and 103 normal samples^19^, was utilized in this study. The clinical features of participants diagnosed with IPF and unaffected controls are summarized in Table 1. Age, gender, ethnicity, and smoking history showed no statistically significant disparities between healthy individuals and IPF patients in this cohort. The GSE110147 dataset contained 22 idiopathic pulmonary fibrosis samples and 11 normal samples^20^. DEGs are identified using the above GEO dataset.

**Table 1:** The clinical characteristics of individuals identified with IPF and unaffected control subjects in GSE150910.

Characteristic	IPF (n = 103)	Control (n = 103)	*p* value
Age, year	60.3 ± 8.3	59.9 ± 10.2	0.98
Sex	n = 103	n = 103	0.23
M	57 (55%)	45 (44%)	
F	46 (45%)	58 (56%)	
Race	n = 101	n = 103	
Non-hispanic white	85 (84%)	87 (84%)	0.55
Hispanic	7 (7%)	4 (4%)	–
Asian	2 (5%)	3 (3%)	–
Black	4 (4%)	9 (9%)	–
Other	3 (3%)	0 (0%)	–
Somke	n = 95	n = 96	0.9
Ever	40 (42%)	43 (45%)	
Never	55 (58%)	53 (55%)	
Sampling method			0.51
Surgical lung biopsy	36 (35%)	41 (40%)	
Transplant	67 (65%)	62 (60%)	

*IPF* idiopathic pulmonary fibrosis. Continuous variables are shown as mean ± SD, and categorical variables are shown as (n%). *p* values are provided among three groups.

### DEGs screening, data processing, and DEG analysis

To remove batch effects from both datasets, we used the merge and combat functions of SVM to create metadata groups. We identified DEGs with the threshold of FDR < 0.05 and |log_2_ foldchange (FC)|> 0.585 between idiopathic pulmonary fibrosis and normal samples via package “DESeq2” in R software (version: 4.1.2). Volcano maps and heat maps of DEGs were visualized using the “pheatmap” (Version:1.0.12) and “ggplot2” (Version:3.4.2) software packages. In the volcano plot, DEGs with log_2_FC < 0 were considered to be down-regulated, while those with log_2_FC > 0 were considered to be up-regulated^21^. The heatmap of the expression of biomarkers are visualized using the “pheatmap” packages (Version:1.0.12).

### Functional enrichment analysis

To explore the underlying mechanisms of DEGs in idiopathic pulmonary fibrosis, the “clusterProfiler” R package^22^ was used to perform the Gene Ontology (GO) and the Kyoto Encyclopedia of Genes and Genomes (KEGG) enrichment analysis^23–25^. Statistical significance was defined as FDR values less than 0.05 for both KEGG and GO enrichment analyses.

### Candidate biomarker screening

In this study, three machine learning algorithms were utilized to screen out characteristic genes of idiopathic pulmonary fibrosis, including random forests (RF), least absolute shrinkage and selection operator (LASSO) logistic regression, and support vector machine-recursive feature elimination (SVM-RFE). It is reported that RF^26, 27^, LASSO logistic regression^17^, and SVM-RFE^28^ algorithms were of great significance to identify key biomarkers. In recent years, these three algorithms have been widely used in research to identify diagnostic or prognostic factors^29–34^. The RF algorithm was performed with package “randomForest” in R software (version: × 64 4.1.2); LASSO logistic regression analysis was carried out with package “glmnet” in R software (version: × 64 4.1.2); SVM-RFE algorithm was performed with package “e107” in R software (version: × 64 4.1.2). The potential biomarkers were yielded by intersecting the characteristic genes identified by RF, LASSO logistic regression, and SVM-RFE algorithms. Furthermore, the accuracy of the biomarkers was evaluated by the receiver operating characteristic (ROC) curve in the training set and test set.

### Assays of immune cellular patterns in microenvironment

CIBERSORT is a deconvolution algorithm used to calculate the abundance of 22 types of infiltrated immune cells between the idiopathic pulmonary fibrosis group and the control group^35^. P less than 0.05 was considered statistically significant. Group comparisons were performed using the Wilcoxon rank sum test. The package “ggplot2” was employed to draw a violin plot for visualize the distinction of immune infiltrating cells. Furthermore, the “corrplot” package was used to visualize the correlation heat map of the relationship between 22 immune infiltrating cells.

### Correlation analysis between biomarkers and infiltrating immune cells

The relationship of the biomarkers with the levels of immune infiltrating cells was explored using Spearman’s rank correlation analysis in R software (version: × 64 4.1.2). The “ggplot2” package was used to visualize the results. *p* values < 0.05 were considered statistically significant^36^.

### Statistical analysis

All statistical analyses were performed using the R software 4.1.2 and GraphPadPrism 8. RF analysis was conducted using the “RandomForest” Package, the LASSO Cox regression was undertaken by the “glmnet” package, and the SVM analysis was done by using the “e1071” R package. ROC curves were used to estimate the diagnostic accuracy of the cancer markers. Spearman's rank correlation test was used to establish the significance of correlation between the expression of genes and the infiltration of immune cells. The wilcoxon test identifies the significance of any differences between the two groups. The statistically significant difference was defined as the p value being less than 0.05.

### Ethics statement

The animal study was reviewed and approved by the Research Ethics Committee of Jiangxi University of Traditional Chinese Medicine.

## Results

### Identification of differentially expressed genes in idiopathic pulmonary fibrosis

**Figure 1:**
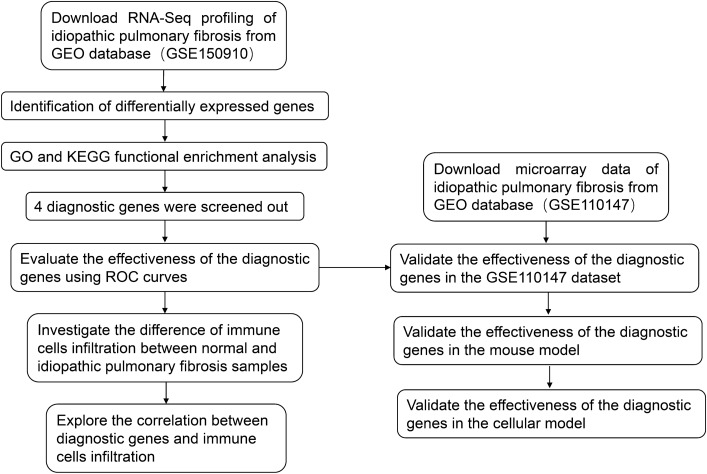
The workflow of this study.

**Figure 2:**
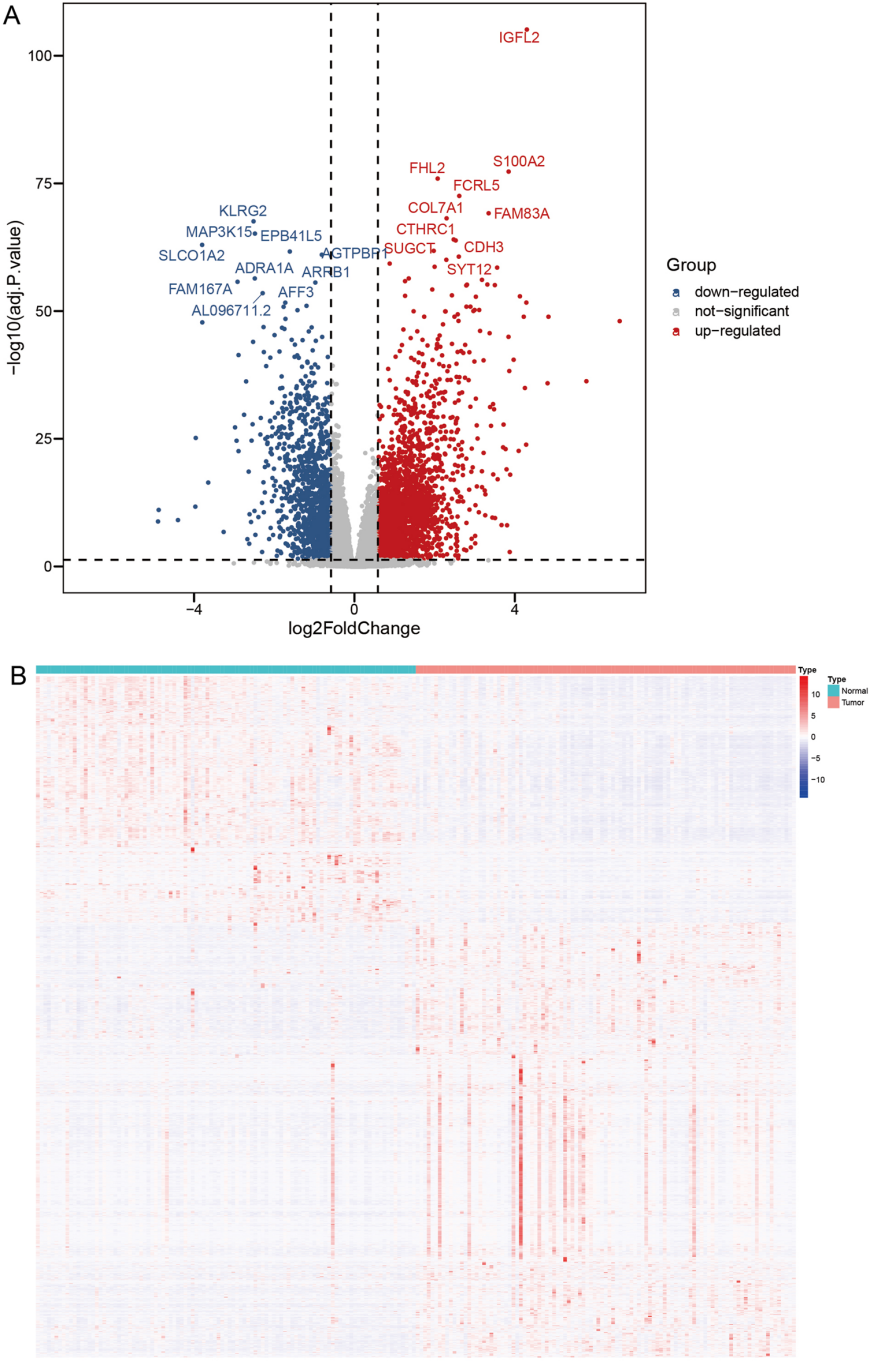
Identification of differentially expressed genes between idiopathic pulmonary fibrosis and normal samples. (**A**) Volcano plot of the GSE150910 dataset with the cut-off criteria of |log_2_FC|> 0.585 and FDR < 0.05. (**B**) Heatmap visualization of the DEGs between idiopathic pulmonary fibrosis and normal samples.

The workflow of this study is illustrated in Fig. 1. A total of 103 normal samples and 103 idiopathic pulmonary fibrosis samples from GSE150910 were used to evaluate the differences between the two samples, including 2359 upregulated genes and 1299 downregulated genes, by identification using the “DESeq2” package (Fig. 2A). The heatmap of these differentially expressed genes was shown in Fig. 2B.

### Functional correlation analysis

**Figure 3:**
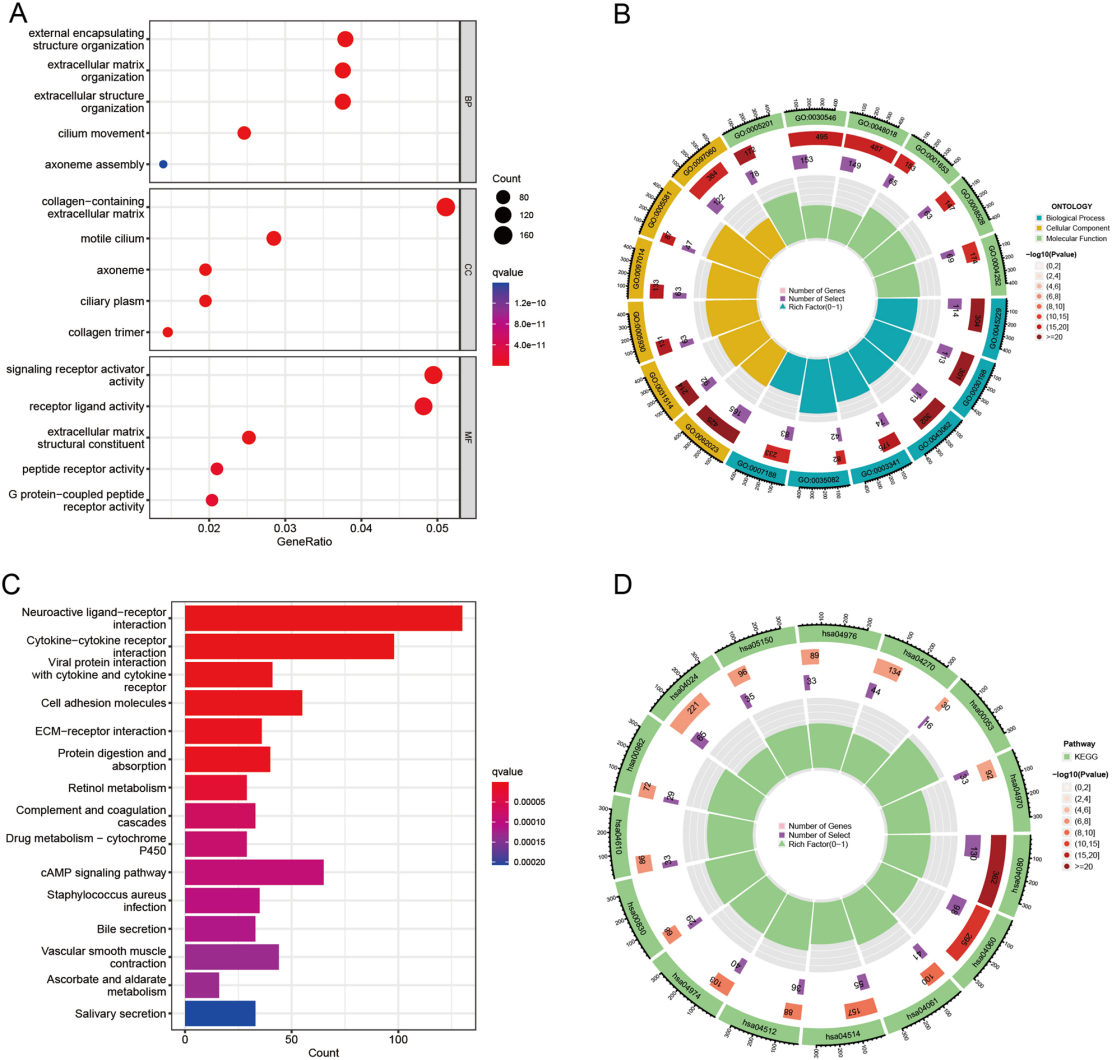
The results of functional enrichment analyses. (**A**,**B**) GO analysis of DEGs. (**C**,**D**) KEGG pathway enrichment analysis of DEGs.

To further explore the potential biological functions of DEGs of idiopathic pulmonary fibrosis in human, we constructed GO and KEGG enrichment analysis The results of GO enrichment analysis suggested that differentially expressed genes were mainly enriched in external encapsulating structure organization, extracellular matrix organization, and extracellular structure organization in the biological process aspect (Figs. 3A,B). In the aspect of cellular components, these differentially expressed genes were mainly involved in collagen–containing extracellular matrix, motile cilium, and axoneme. In the aspect of molecular function, these differentially expressed genes were mainly gathered in signaling receptor activator activity, receptor ligand activity, and extracellular matrix structural constituent. KEGG pathway enrichment analysis revealed that the differentially expressed genes were mainly enriched in 15 pathways, such as Neuroactive ligand–receptor interaction, Cytokine–cytokine receptor interaction, and Viral protein interaction with cytokine and cytokine receptor (Figs. 3C,D). These results suggest that extracellular matrix plays an important role in idiopathic pulmonary fibrosis.

### Identification and assessment of biomarkers

**Figure 4:**
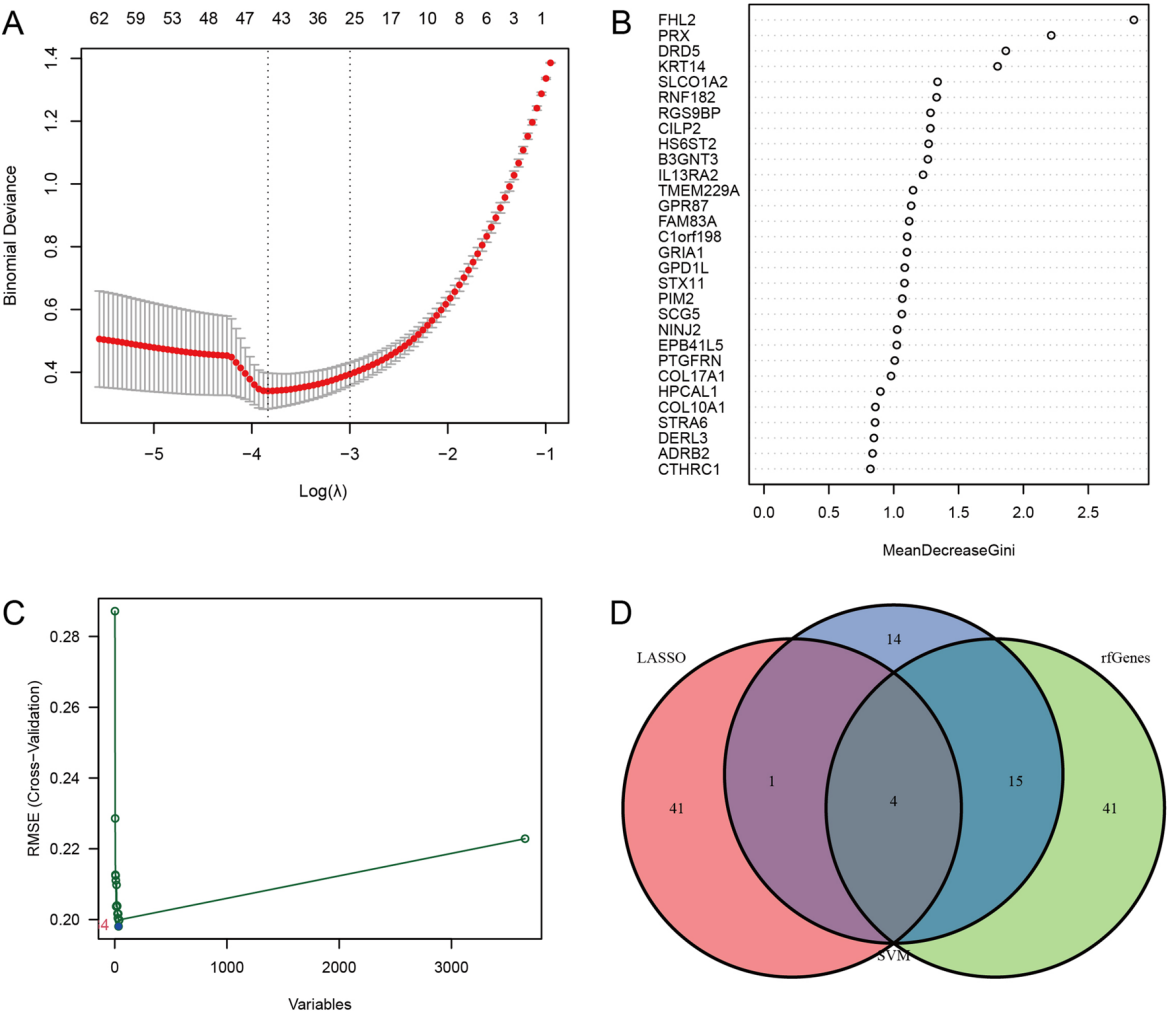
Identification of biomarkers of idiopathic pulmonary fibrosis. (**A**) Characteristic genes selection via LASSO algorithm. (**B**) Characteristic genes selection via random forest algorithm. (**C**) Characteristic genes selection via SVM-RFE algorithm. (**D**) Venn diagram showed the intersection of characteristic genes obtained by the three indicated algorithms. The overlapping characteristic genes represent the biomarkers of idiopathic pulmonary fibrosis.

**Figure 5:**
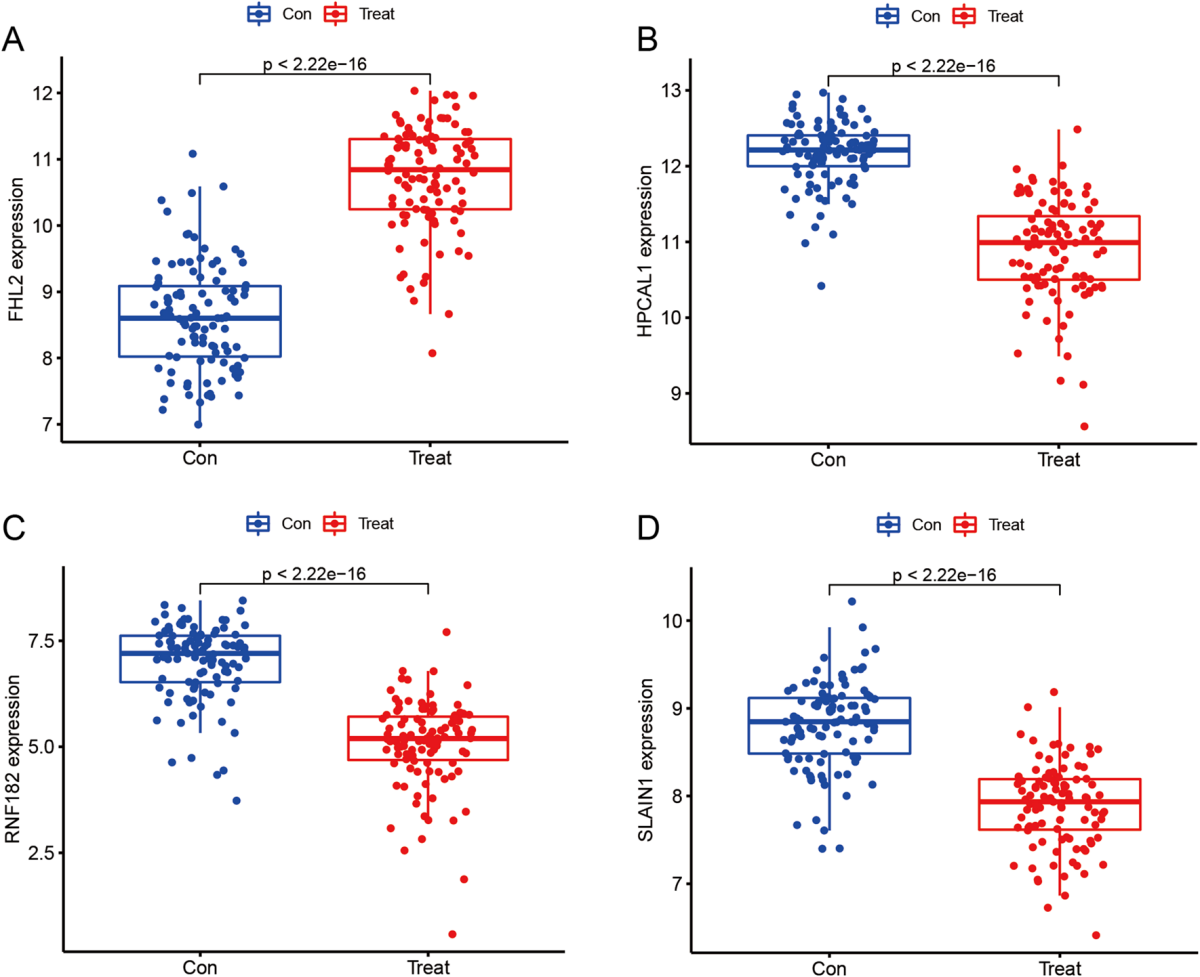
Box plots of the expression of biomarkers between idiopathic pulmonary fibrosis and normal samples in the training set, including (**A**) FHL2, (**B**) HPCAL1, (**C**) RNF182, and (**D**) SLAIN1.

**Figure 6:**
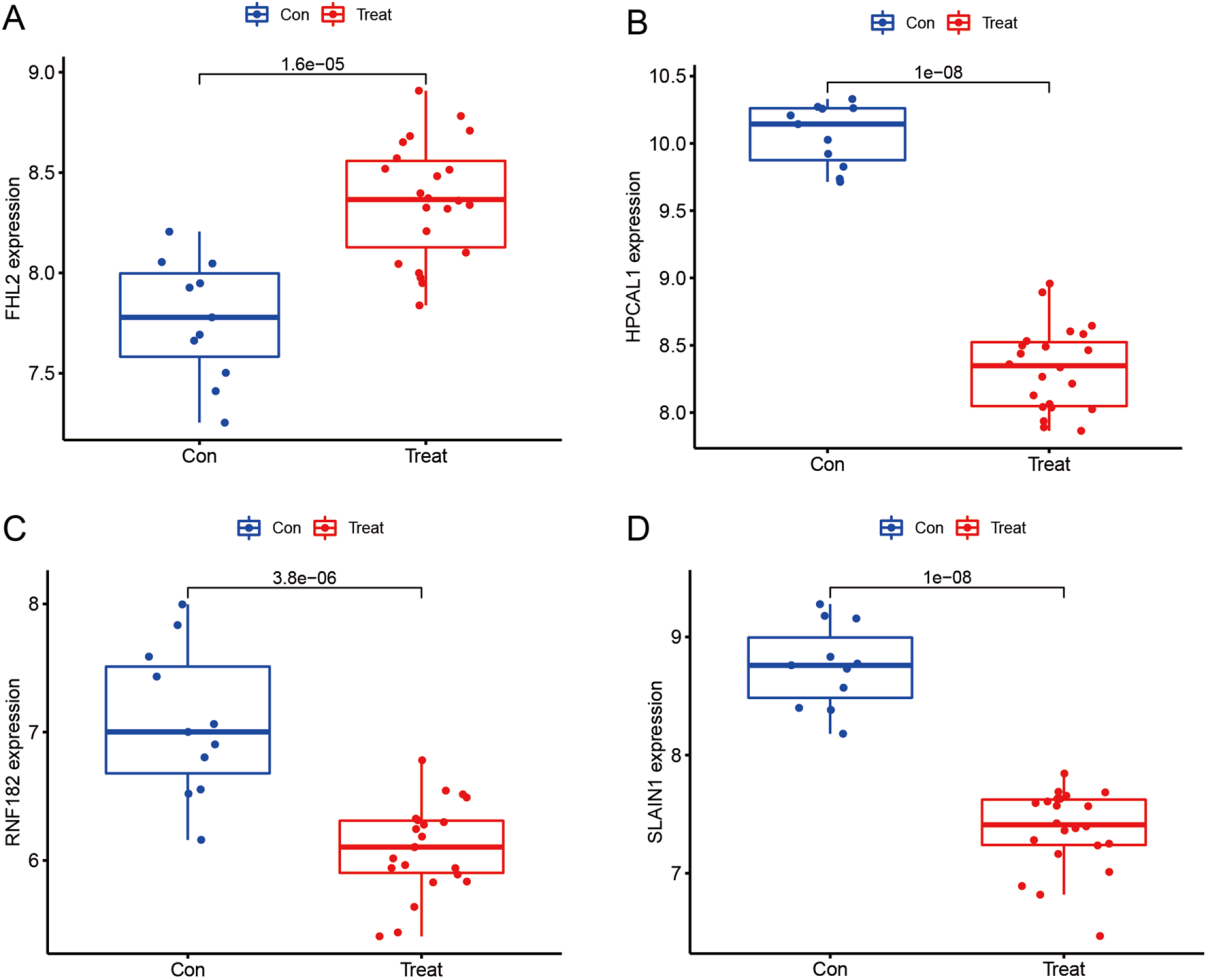
Box plots of the expression of biomarkers between idiopathic pulmonary fibrosis and normal samples in the test set, including (**A**) FHL2, (**B**) HPCAL1, (**C**) RNF182, and (**D**) SLAIN1.

**Figure 7:**
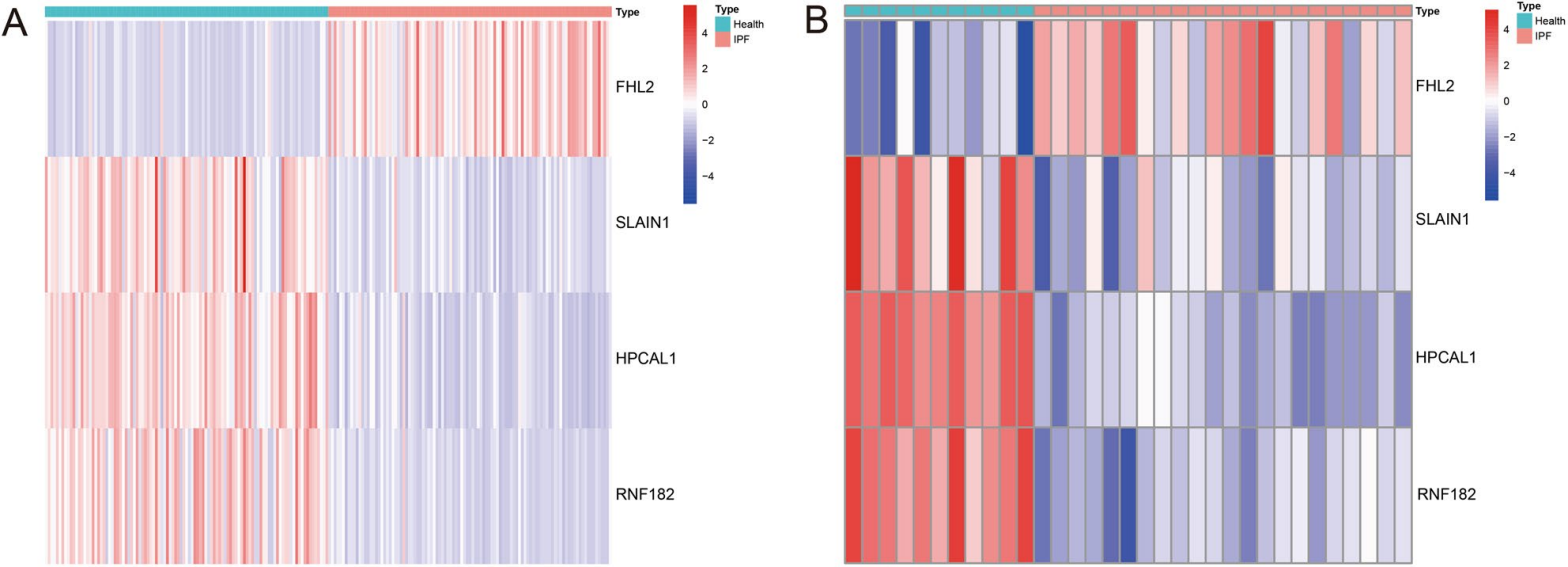
Heatmap of the four biomarkers in the training set (**A**) and test set (**B**).

Three validated machine learning algorithms (LASSO, RF, SVM-RFE) were applied to identify key characteristic genes associated with IPF. 46 characteristic genes were identified using LASSO algorithm (Fig. 4A). 60 characteristic genes were screened out using RF algorithm (Fig. 4B). Moreover, 34 characteristic genes were identified as biomarkers based on SVM-RFE algorithm (Fig. 4C). Only the overlapping genes (FHL2, HPCAL1, RNF182 and SLAIN1) were ultimately selected as biomarkers of IPF (Fig. 4D). In addition, the selected biomarkers showed good differential expression in the training set and test set, the expression levels of FHL2 were elevated in the idiopathic pulmonary fibrosis group (Fig. 5A), while the expression level of SLAIN1, HPCAL1, and RNF182 were reduced in the idiopathic pulmonary fibrosis group (Fig. 5B–D). As indicated by the differential analysis in the test set, the results were consistent with the outcomes of the training set (Fig. 6A–D). The heatmap of these four biomarkers in the training set and test set were shown in Fig. 7A–B, which suggested that the expression level of FHL2 was correlated with IPF group positively, the expression level of SLAIN1, HPCAL1, and RNF182 were correlated with IPF group negatively.

### Diagnostic effectiveness of biomarkers in idiopathic pulmonary fibrosis

To further assess the diagnostic value of the identified genes in idiopathic pulmonary fibrosis, ROC analysis was conducted for the four key genes in both the training and test sets. The results demonstrated that the four diagnostic biomarkers, as identified by the machine learning algorithm, exhibited strong diagnostic capabilities in the training set. FHL2 had an AUC of 0.954 (95% CI 0.924–0.978), HPCAL1 had an AUC of 0.955 (95% CI 0.926–0.979), RNF182 had an AUC of 0.917 (95% CI 0.875–0.955), and SLAIN1 had an AUC of 0.916 (95% CI 0.874–0.954) (Supplementary Fig. 1A–D). Furthermore, the diagnostic effectiveness of these biomarkers was validated in the independent test set, with FHL2 achieving an AUC of 0.926 (95% CI 0.822–0.992), HPCAL1 an AUC of 1.000 (95% CI 1.000–1.000), RNF182 an AUC of 0.946 (95% CI 0.843–1.000), and SLAIN1 an AUC of 1.000 (95% CI: 1.000–1.000) (Supplementary Fig. 2A–D). As illustrated in the figures above, all four genes exhibited strong discriminatory ability for idiopathic pulmonary fibrosis.

### Evaluation of SLAIN1 expression in vivo and in vitro

**Figure 8:**
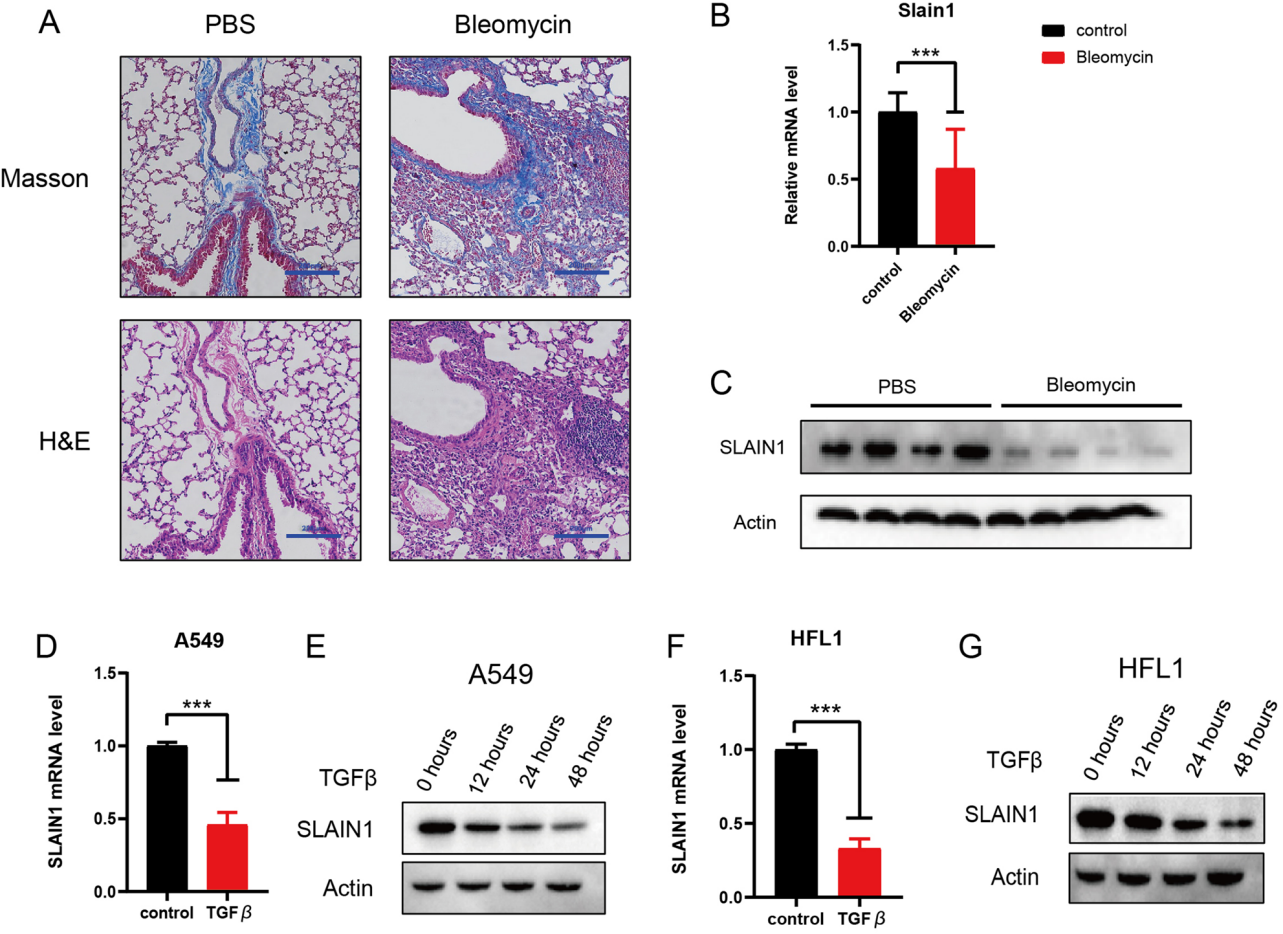
Validation the role of SLAIN1 in vivo and vitro*.* (**A**) Photomicrographs of PBS-treated lung sections and Bleomycin-treated lung sections stained with Masson staining and HE staining, respectively. (**B**) Quantification of mRNA expression levels of SLAIN1 in the mouse model. (**C**) Western blot for SLAIN1 in the mouse model. Bleomycin-treated group was the experimental group, and PBS-treated group was the control group. (**D**) Quantification of SLAIN1 expression level in the A549 cells. (**E**) Western blot of SLAIN1 in A549 cells over time with fibrosis. (**F**) Quantification of SLAIN1 expression level in the HFL1 cells. (**G**) Western blot of SLAIN1 in HFL1 cells over time with fibrosis.

To ensure the robustness of our findings, we initiated our investigation by establishing a murine model and a cellular model. As shown in Fig. 8A, Masson and HE staining revealed that the pulmonary fibrosis in bleomycin-treated mice lung tissues was notably more severe than in the PBS-treated mice lung tissues, thus affirming the successful construction of the mouse model. Subsequently, our attention turned to examining the expression level of SLAIN1, which was found to be significantly lower in the IPF samples compared to the normal samples (Fig. 8B,C).

Furthermore, we extended our investigation to evaluation the expression level of SLAIN1 in A549 and HFL1 cells. To replicate the conditions of IPF in vitro, we exposed A549 and HFL1 cells to TGF-β. As depicted in Fig. 8D,F, the mRNA expression level of SLAIN1 in A549 and HFL1 cells was substantially reduced. Moreover, western blotting demonstrated a gradual decrease in the expression level of SLAIN1 in A549 and HFL1 cells (Fig. 8E,G). In summary, our comprehensive examination of SLAIN1 expression both in vivo and in vitro strengthens our understanding of its potential role as a biomarker and its involvement in the pathogenesis of idiopathic pulmonary fibrosis.

### Immune infiltration

The infiltration status of 22 types of immune cells between idiopathic pulmonary fibrosis group and control group were assessed with CIBERSORT algorithm. The percentage of the 22 types of immune cells between idiopathic pulmonary fibrosis group and control group was shown in the bar plot (Supplementary Fig. 3A). The correlation of 22 types of immune cells revealed that T cells follicular helper was positively related with Plasma cells (r = 0.41), NK cells resting was positively related with T cells CD4 naive (r = 0.37), whereas T cells follicular helper was negatively related to T cells CD4 memory resting (r =− 0.51), NK cells resting was positively related with T cells follicular helper (r =− 0.39) (Supplementary Fig. 3B). The violin plot of the immune cell infiltration difference demonstrated that patients with idiopathic pulmonary fibrosis had a higher level of B cells memory, Plasma cells, T cells CD8, T cells follicular helper, T cells regulatory (Tregs), Macrophages M0, and Mast cells resting compared with the control group (Supplementary Fig. 3C).

### Correlation analysis between biomarkers and immune cells

As indicated from the correlation analysis, SLAIN1 displayed a positive correlation with Eosinophils (r = 0.26, *p* < 0.001), Monocytes (r = 0.47, *p* < 0.001), Neutrophils (r = 0.22, *p* < 0.05), NK cells resting (r = 0.49, *p* < 0.001), T cells CD4 memory resting (r = 0.45, *p* < 0.001), and T cells CD4 naïve (r = 0.2, *p* < 0.05), and a negative correlation with B cells memory (r =− 0.28, *p* < 0.001), B cells naïve (r =− 0.2, *p* < 0.05), Macrophages M0 (r =− 0.19, *p* < 0.05), Mast cells activated (r =− 0.18, *p* < 0.05), NK cells activated (r =− 0.15, *p* < 0.05), Plasma cells (r =− 0.53, *p* < 0.001), T cells CD8(r =− 0.15, *p* < 0.05), T cells follicular helper (r =− 0.43, *p* < 0.001), and T cells regulatory (Tregs) (r =− 0.39, *p* < 0.001) (Supplementary Fig. 4A–J). It can be concluded that SLAIN1 was correlated with immune cells.

## Discussion

When the lung sustains injury, the fibrosis process begins and the disease continues to progress^37, 38^. Idiopathic pulmonary fibrosis is a lethal, progressive fibrosing parenchymal lung disease that affects millions of patients worldwide and is refractory to most treatment options^39^. Fibrosis may play an important role in the later development of the disease, which may be the result of the interaction between multiple pathogenic factors^40^. Potentially powerful noninvasive biomarkers can provide critical diagnostic information while also being critical to understanding the course of IPF^41^. Patients at increased risk for IPF may benefit from clinical trials that allow physicians to select credible fibrosis biomarkers^42^.

In this study, we downloaded two GSE datasets from the GEO database to identify differentially expressed genes between IPF and normal lung tissues. Next, GO and KEGG analyses were then performed to explore the biological functions of DEGs in IPF. Combined with least absolute shrinkage and selection operator (LASSO) logistic regression, support vector machine-recursive feature elimination (SVM-RFE), and random forest (RF) algorithms, four biomarkers for idiopathic pulmonary fibrosis were screened out. The receiver operating characteristic (ROC) curve was calculated to additionally evaluate the diagnostic accuracy of biomarkers. Moreover, the CIBERSORT algorithm was employed to assess the infiltration of immune cells and the relationship between the infiltrating immune cells and the biomarkers. Finally, we detected the expression level of SLAIN1 to assess its potential role in the pathogenesis of idiopathic pulmonary fibrosis using a mouse model and cellular model.

As for the four biomarkers, previous studies showed that FHL2 has been identified as a biomarker of lung cancer and elevated level of FHL2 exacerbates the outcome of patients with non-small cell lung cancer (NSCLC) and the malignant phenotype in NSCLC cells^43^. Among visinin-like proteins (VILIPs), HPCAL1 belongs to the neuronal calcium sensor (NCS) family, which is responsible for calcium signaling in neurons^44^. Innate immune responses triggered by TLRs are inhibited by RNF182 by promoting degradation of p65 via K48-linked ubiquitination^45^. SLAIN1, indispensable for neuronal sprouting and brain development, significantly contributes to axon elongation by promoting microtubule growth^46^. Recent findings have suggested that SLAIN1 may play a pivotal role in intellectual disability^47^. Moreover, both in the training set and test set, the prognostic value of these four biomarkers were higher. Therefore, the model we established has excellent accuracy both in the training set and test set. Taken together, these four biomarkers are expected to be potential targets for the diagnosis of idiopathic pulmonary fibrosis.

Furthermore, we confirmed the expression level of SLAIN1 in the pathogenesis of idiopathic pulmonary fibrosis using both a mouse model. Masson and HE staining showed that the pulmonary fibrosis of bleomycin-treated mice lung tissues was more severe than PBS-treated mice lung tissues, which demonstrated that the mouse model was successfully constructed. The SLAIN1 expression level in A549 and HFL1 cells was significantly lower, which indicated that our model was equally successful in cell validation. Based on the above results, we can affirm that our findings bolster the reliability of the prognostic model.

Despite these advantages, this study has some limitations. The molecular mechanisms underlying the identified biomarkers in idiopathic pulmonary fibrosis (IPF) have not been fully investigated, warranting further experimental exploration. Moreover, as clinical patients were not included in this study, the diagnostic potential of the identified genes for IPF was indirectly assessed. Additional prospective studies are necessary to validate and translate these findings into clinical practice.

## Conclusion

In summary, this research successfully pinpointed four promising biomarkers (FHL2, HPCAL1, RNF182, and SLAIN1) and investigated the potential involvement of SLAIN1 in the pathogenesis of idiopathic pulmonary fibrosis. These discoveries hold substantial importance in advancing our comprehension of the disease's mechanisms and identifying potential avenues for therapeutic intervention in idiopathic pulmonary fibrosis.

### Supplementary Information

Supplementary Legends.

Supplementary Figure 1.

Supplementary Figure 2.

Supplementary Figure 3.

Supplementary Figure 4.

Supplementary Figure 5.

## Data Availability

The GSE150910 and GSE110147 datasets used in current study are available in the GEO repository (http://www.ncbi.nlm.nih.gov/geo/). Further inquiries can be directed to the corresponding author.
