# Supplementary material for: Identifying potential biomarkers of idiopathic pulmonary fibrosis through machine learning analysis
Source: Sci Rep. 2023 Oct 2;13:16559. doi: 10.1038/s41598-023-43834-z (PMC10545744; doi:10.1038/s41598-023-43834-z)
Supplement: Supplementary file 1 — Supplementary Legends. [file 41598_2023_43834_MOESM1_ESM.docx]

**Supplementary Figure 1.** ROC curves of the four biomarkers in the training set, including (A) FHL2, (B) HPCAL1, (C) RNF182, and (D) SLAIN1.

**Supplementary Figure 2.** Evaluation of the effectiveness of the four biomarkers in the test set. ROC curves of (A) FHL2, (B) HPCAL1, (C) RNF182, and (D) SLAIN1.

**Supplementary Figure 3.** Visualization of immune cells infiltration in idiopathic pulmonary fibrosis and normal samples. (A) The proportion of infiltrating immune cells in idiopathic pulmonary fibrosis and normal samples. (B) Correlation heatmap of 22 types of immune cells. (C) Violin diagram of the proportion of 22 types of immune cells.

**Supplementary Figure 4.** Correlations between FHL2 and nine infiltrating immune cells in idiopathic pulmonary fibrosis, including (A) Macrophages M0, (B) Mast cells resting, (C) Monocytes, (D) Neutrophils, (E) NK cells resting, (F) Plasma cell, (G) T cells CD4 memory resting, (H) T cells CD4 naïve, (I) T cells follicular helper, and (J) T cells regulatory.
