# Supplementary figures and images for: Identifying potential biomarkers of idiopathic pulmonary fibrosis through machine learning analysis
Source: Sci Rep. 2023 Oct 2;13:16559. doi: 10.1038/s41598-023-43834-z (PMC10545744; doi:10.1038/s41598-023-43834-z)

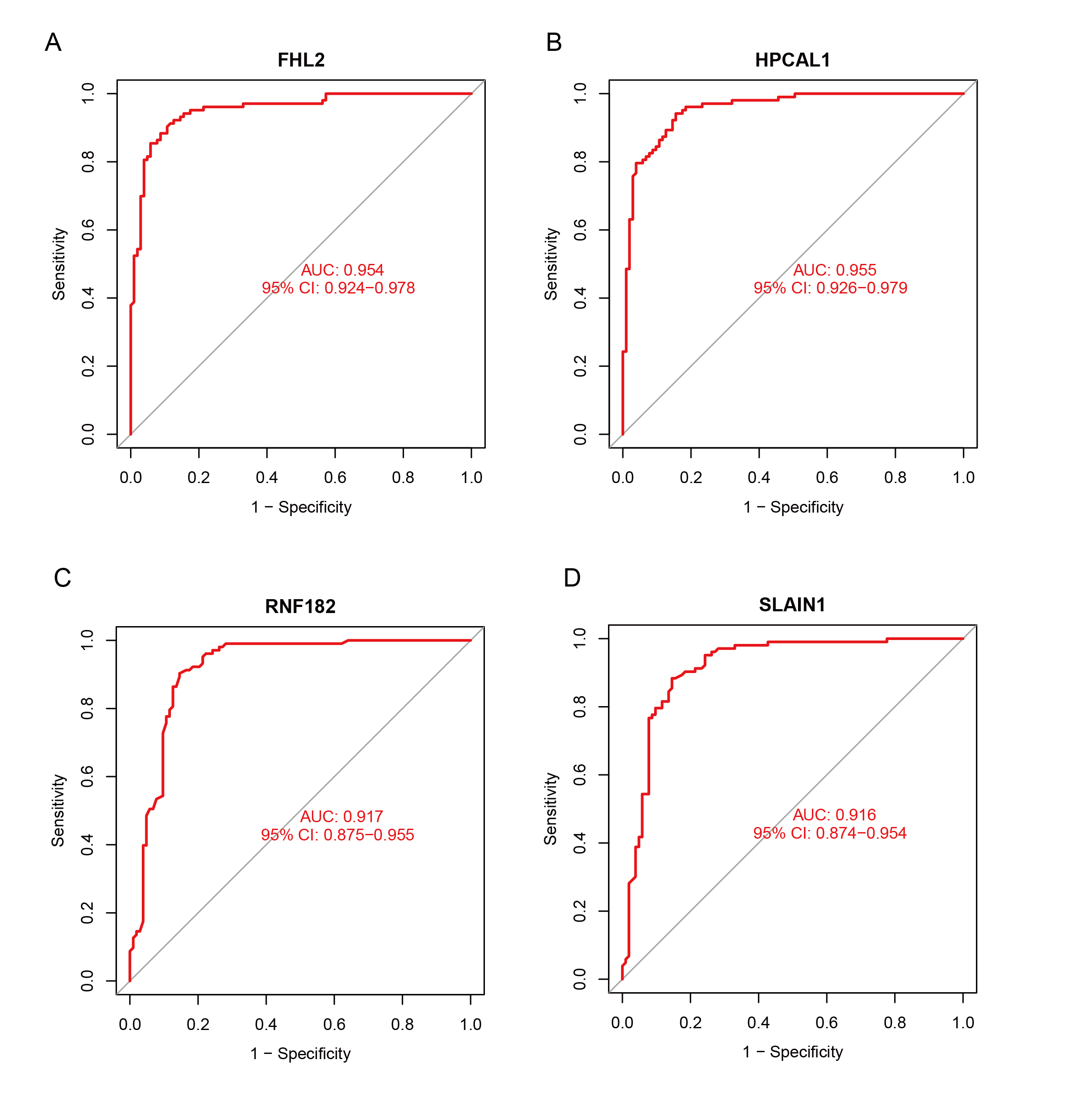

Supplement: Supplementary file 2 — Supplementary Figure 1. [file 41598_2023_43834_MOESM2_ESM.jpg]

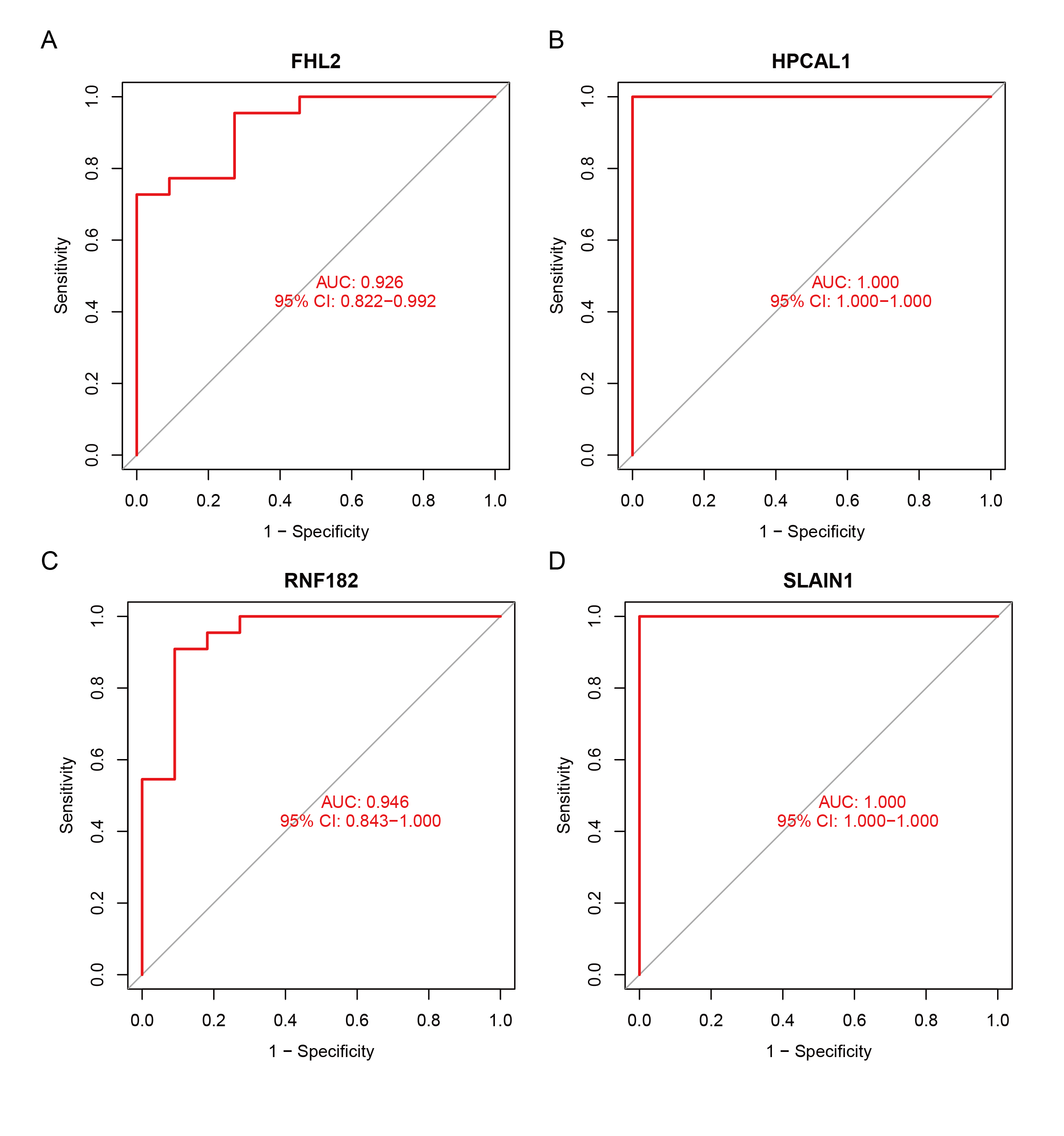

Supplement: Supplementary file 3 — Supplementary Figure 2. [file 41598_2023_43834_MOESM3_ESM.jpg]

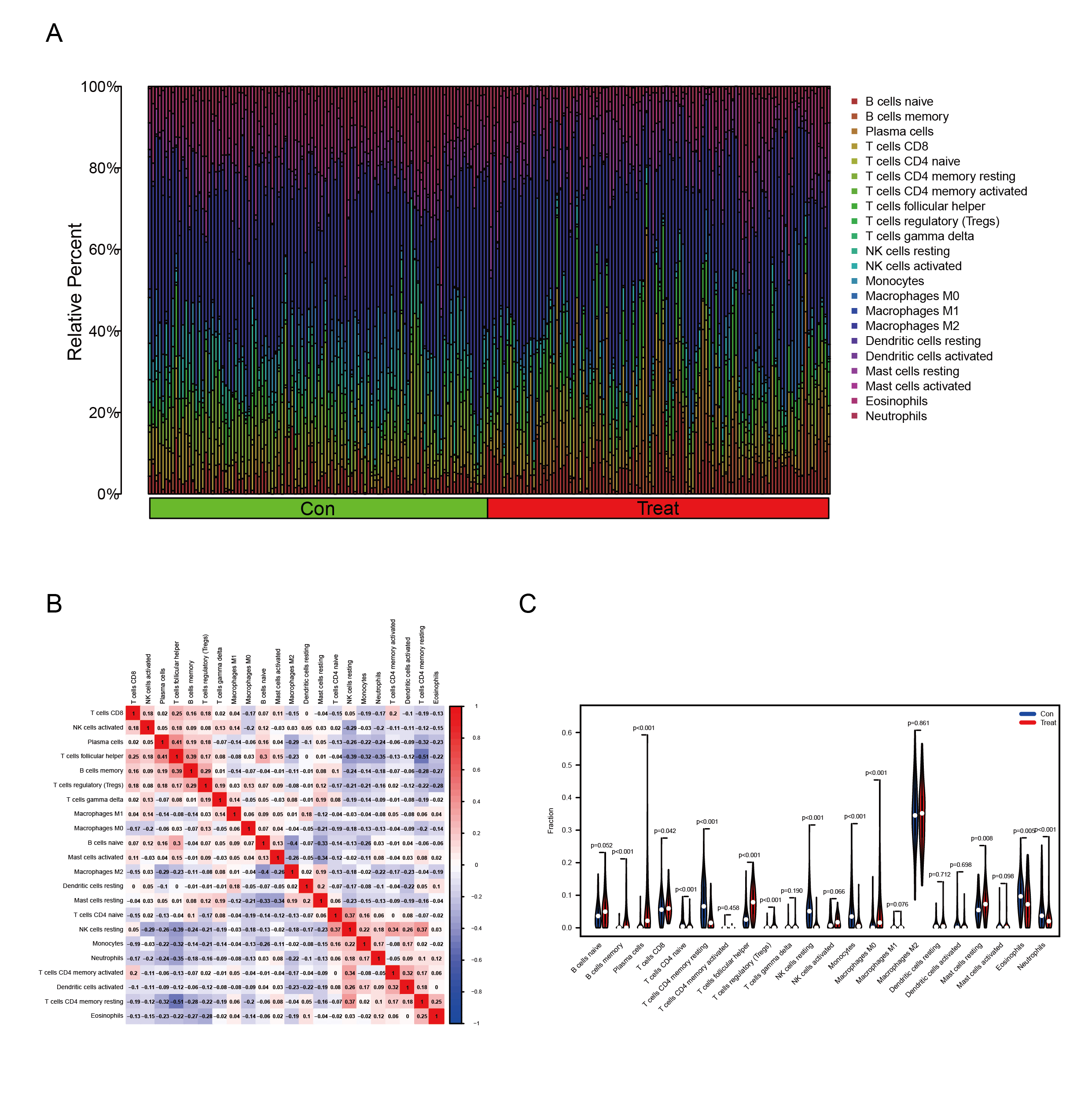

Supplement: Supplementary file 4 — Supplementary Figure 3. [file 41598_2023_43834_MOESM4_ESM.jpg]

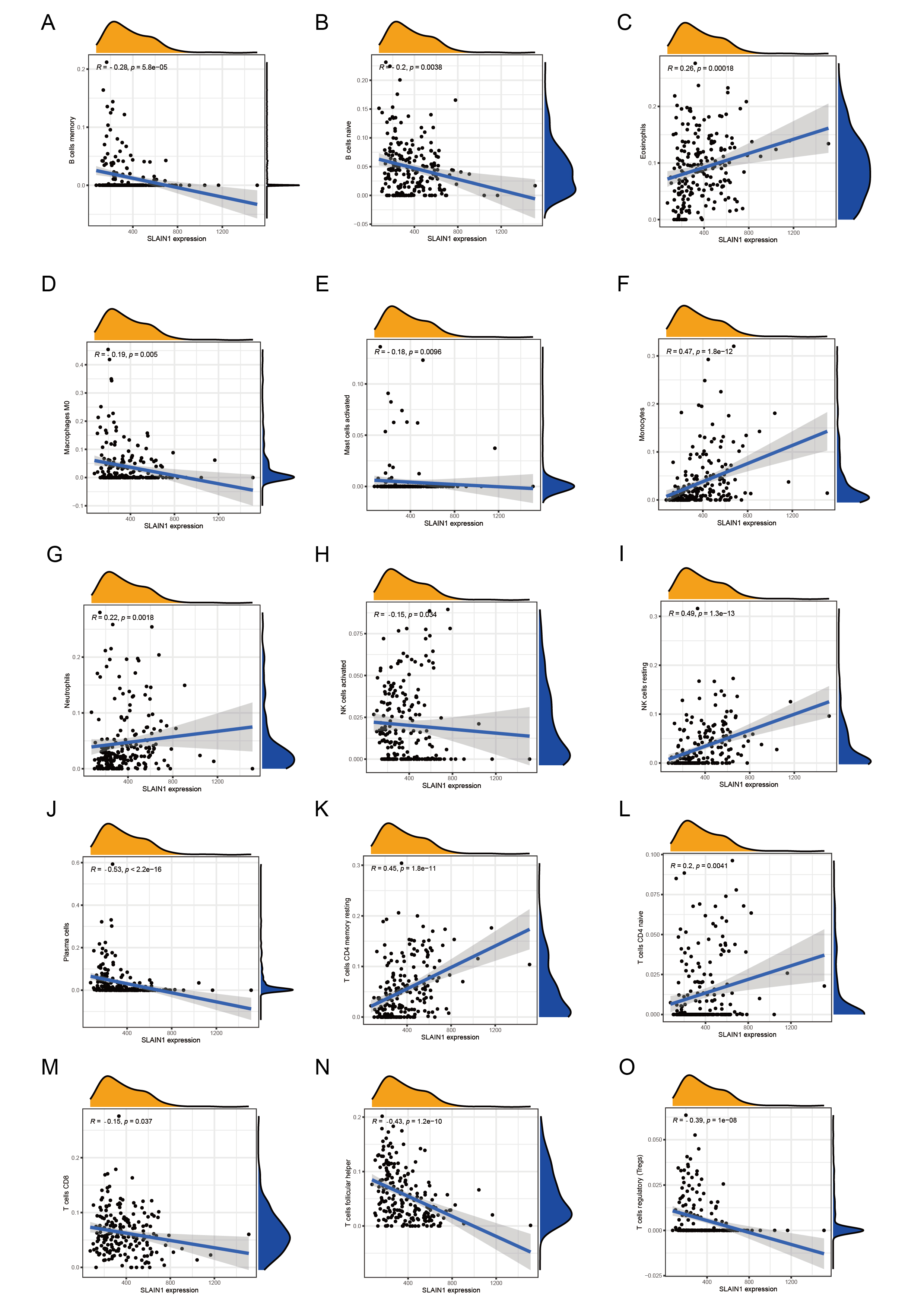

Supplement: Supplementary file 5 — Supplementary Figure 4. [file 41598_2023_43834_MOESM5_ESM.jpg]

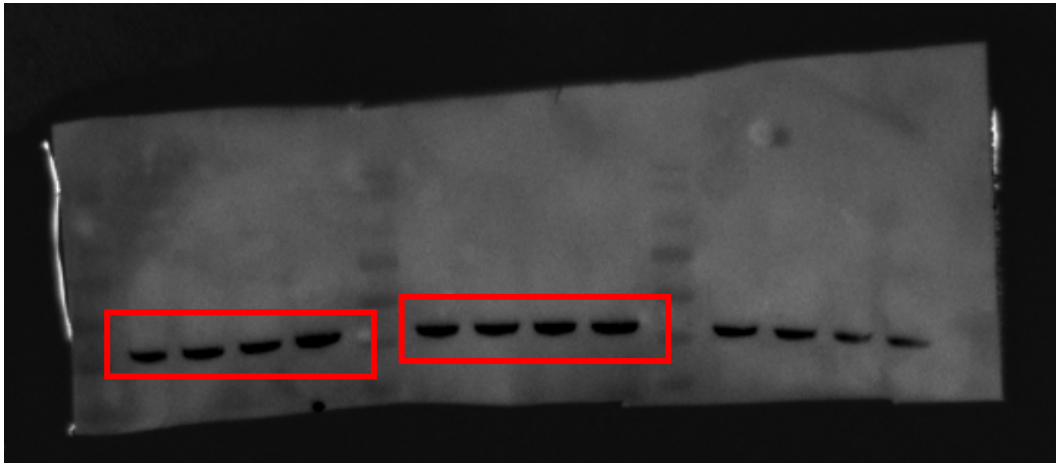

Anti-Actin

Anti-Actin

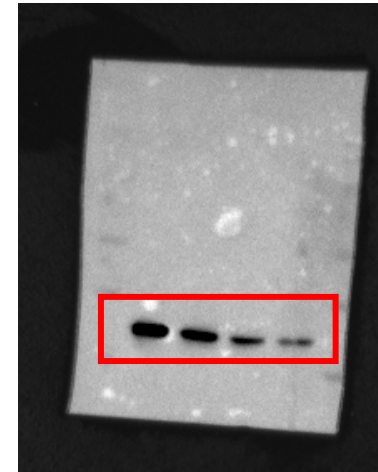

Anti-SLAIN1

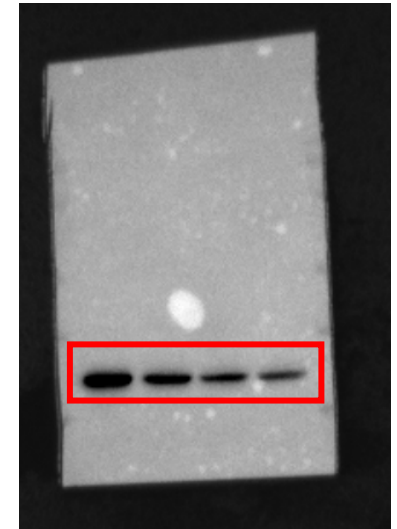

Anti-SLAIN1

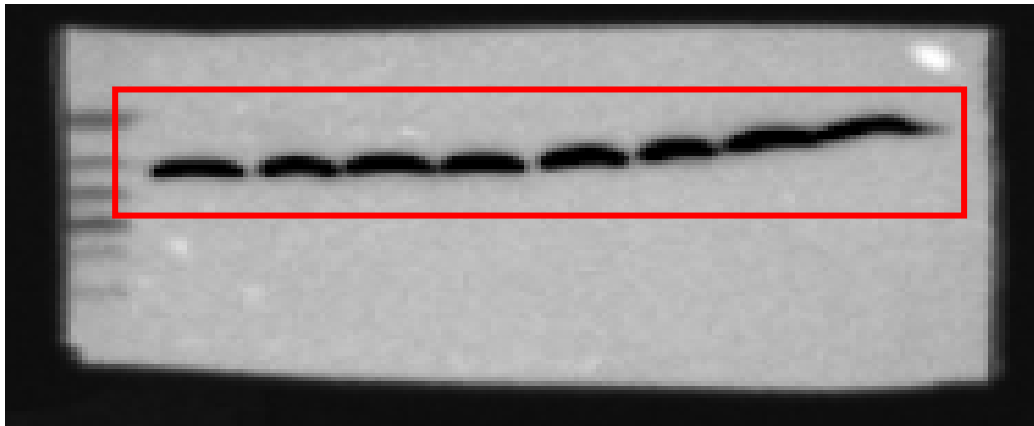

Anti-Actin

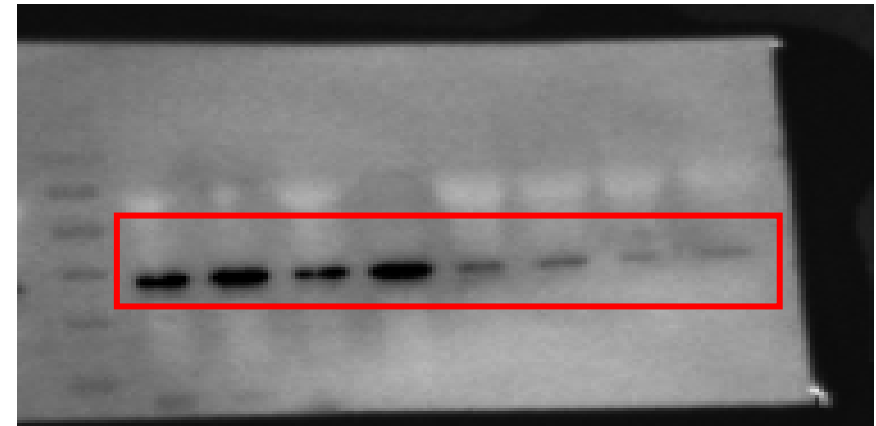

Anti-SLAIN1

Supplement: Supplementary file 6 — Supplementary Figure 5. [file 41598_2023_43834_MOESM6_ESM.pdf]
